# Effects of a Drying Treatment on the Mechanical Properties and Hemodynamic Characteristics of Bovine Pericardial Bioprosthetic Valves

**DOI:** 10.3390/jfb16120434

**Published:** 2025-11-25

**Authors:** Xuan Hu, Zhaoming He, Hao Wang

**Affiliations:** 1 Research Center of Fluid Machinery Engineering and Technology, Jiangsu University, Zhenjiang 212013, China; 18076091178@163.com; 2 Department of Mechanical Engineering, Texas Tech University, Lubbock, TX 79409, USA; 3 School of Electrical and Information Engineering, Jiangsu University, Zhenjiang 212013, China

**Keywords:** bovine pericardium, drying treatment, anisotropy, constitutive model

## Abstract

The high incidence of cardiovascular disease and the early failure of bioprosthetic valves due to calcification have driven the development of anti-calcification technologies. As a new storage technology, drying treatment is expected to delay the calcification process by reducing glutaraldehyde residues. However, the effects of drying treatment on the mechanical properties and valve functions of bovine pericardial materials are still unclear. The objective of this study is to evaluate the influence of drying and rehydration treatments on the mechanical integrity and geometric properties of bovine pericardium and the hemodynamic performance of bioprosthetic valves made with these tissues. Cross-linked bovine pericardial samples (*n* = 15) were divided into three groups—wet (control group progressed with normal glutaraldehyde), dehydrated (ethanol–glycerol dehydration), and rehydration (saline immersion) groups—and the geometric stability and nonlinear mechanical behaviors of the materials were analyzed via thickness measurements and uniaxial and biaxial tensile tests. Quantitative results showed that thickness remained stable across groups (wet: 0.356 ± 0.052 mm; dry: 0.361 ± 0.053 mm; rehydrated: 0.361 ± 0.053 mm, *p* > 0.05). Elastic modulus values were preserved (wet: 12.5 ± 1.8 MPa; dry: 13.1 ± 2.0 MPa; rehydrated: 12.7 ± 1.9 MPa, *p* > 0.05), and anisotropy ratio showed no significant changes (1.53 ± 0.06 vs. 1.57 ± 0.07, *p* > 0.05). The hemodynamic performance of bioprosthetic valves made with these materials was evaluated in vitro using a pulsating flow simulation. Hemodynamic parameters demonstrated excellent preservation: effective orifice area (wet: 2.625 ± 0.11 cm^2^; rehydrated: 2.585 ± 0.12 cm^2^, Δ = 1.5%, *p* = 0.32) and regurgitation fraction (wet: 39.35 ± 2.9%; rehydrated: 42.78 ± 3.2%, *p* = 0.15) showed no statistically significant differences. The geometric properties of the material were not significantly changed by the drying treatment, and the material maintained its nonlinear viscoelastic characteristics and anisotropy. The rehydrated bioprosthetic valves did not differ significantly from those in the wet group in terms of the effective orifice area, regurgitation fraction, and transvalvular pressure difference, and the hemodynamic performance remained stable.

## 1. Introduction

Bioprosthetic heart valves have been extensively used due to the improved durability imparted by tissue-processing techniques, especially in minimally invasive heart valve replacement. Pericardial tissue treated with glutaraldehyde cross-linking is now the most popular material used in bioprosthetic heart valves and surgical operations [1,2]. However, its durability is compromised by calcification and panus growth [3,4]. A promising new tissue treatment technique, dry storage, has recently shown good clinical application prospects [5,6]. 

Drying blocks calcium ion binding sites through physical dehydration, which significantly reduces the risk of calcification and provides multiple advantages [7,8]. First, the dried material can be stored at room temperature without chemical preservatives, which greatly improves the convenience of storage [9]. Second, dried bioprosthetic valves are more suitable for the development of pre-assembled systems, which can meet the needs of rapid implantation in emergency situations [6,10]. Furthermore, preliminary results from animal models have shown that the rate of valve calcification after drying treatment was reduced by 40% compared with traditional GA treatment [4,11]. Although drying has shown good potential in preliminary studies, the resulting biomechanical properties have not been fully evaluated, especially regarding [1] the effects of dehydration and rehydration processes on the collagen ultrastructure, the change in fatigue performance under long-term cyclic loading, and hemodynamic equivalence with traditional wetstored valves [12,13].

The mechanical properties of biological valve materials are some of the core factors that determine their clinical durability [14,15]. Bovine pericardium has become the preferred material for aortic and pulmonary valve replacement due to its excellent biocompatibility and mechanical strength [6]. Extensive studies have conducted biaxial tensile testing on wet bovine pericardium [14]. However, the biaxial tensile behavior of hydrated bovine pericardium has not been systematically determined. The drying process may lead to the dehydration and shrinkage of collagen fibers and change the slip characteristics between fibers, thus affecting the material’s anisotropy under biaxial loading. The damage threshold of collagen fibers in the hydrated pericardium reported in [3] was 40% lower than that in wet pericardium, suggesting that drying may amplify the risk of calcification induced by mechanical stress; however, the biaxial loading scenario was not considered [16,17]. 

The aim of this study was to systematically evaluate the effects of a drying treatment on the geometric characteristics, mechanical properties, and hemodynamic performance of bovine pericardial bioprosthetic valves. Specifically, this study focused on quantifying the impact of dehydration and rehydration processes on tissue ultrastructure, determining key geometric and mechanical characteristics that are critical to valve fit and function, and establishing hemodynamic equivalence between dried and traditional preservation methods through comprehensive in vitro testing.

## 2. Materials and Methods

### 2.1. Acquisition and Processing of Bovine Pericardium

The valve leaflets were prepared in our lab from glutaraldehyde-treated bovine pericardium (Shanghai Wilfer Medical Co., Shanghai China, 0.4 mm thick). A trileaflet frame was designed and 3D printed locally to match the dimensions of a standard aortic valve. We did not use any commercial Edwards components. This setup allowed us to maintain full control over geometry and material uniformity.

### 2.2. Combined Dehydration and Rehydration Process

Definition of Terms: In this study, hydration refers to the process of absorbing water into the tissue; drying refers to the removal of water content from the tissue; and rehydration involves the process of reintroducing water to the dried tissue.

The bovine pericardium was treated with GA, placed in a container with normal saline (0.1 M, pH 7.4), and gently agitated using forceps to remove any glutaraldehyde remaining on the pericardial surface. The saline was changed, and rinsing was performed 2–3 times until there was no obvious residue on the pericardial surface. The cleaned bovine pericardium was placed on a dust-free cloth and another dust-free cloth was used to gently press the surface, repeatedly dabbing excess water to ensure that the surface of the pericardium was hydrated without obvious water traces.

Standard Environmental Conditions: All experimental procedures were conducted under controlled laboratory conditions (temperature: 23 ± 1 °C, relative humidity: 50 ± 5% RH) to ensure consistent dehydration conditions and reproducibility. The dried bovine pericardium was soaked in ethanol, with the volume of alcohol and the mass of the pericardium configured in standard proportions. The soaked pericardium was placed on an oscillator and shaken at a constant speed for 10 min to promote initial dehydration. The pericardium was then removed from the ethanol and the residual solution was wiped off using an alcohol-free cloth. The pericardium was re-immersed in the glycerol complex solution, with the volume of the solution configured in proportion. The pericardium was oscillated on an oscillator for 2 h to ensure adequate internal dehydration of the pericardium. After the oscillation was complete, the pericardium was removed from the glycerol solution, and the residual liquid on the pericardial surface was carefully wiped off using a dust-free cloth. After drying, the pericardium was sealed in a clean, sterile plastic bottle to prevent moisture absorption and contamination. 

### 2.3. Rehydration Process

The dried pericardium was soaked in normal saline for 10 min to promote rehydration at a rate of 80%; after it had completely absorbed the water, its soft state was restored. The rehydration process was performed under standardized laboratory conditions with careful monitoring of tissue hydration levels using gravimetric analysis.

### 2.4. Measurement of the Bovine Pericardial Geometry

The thickness of the pericardium was measured using a three-point contact measurement method with a digital micrometer from Shanghai Mitutoyo Medical (Shanghai, China, with an accuracy of ± 0.01 mm). The fifteen pretreated bovine pericardial specimens (10 × 10 mm^2^) were placed in a standard temperature and humidity environment (23 ± 1 °C, 50 ± 5% RH) for 24 h, and three independent measurements were carried out according to the ISO 4593 standard [18]. The arithmetic mean value was taken as the nominal thickness of the sample, and abnormal samples with a thickness coefficient of variation >5% were excluded. A winding curve test was performed for flexure measurements utilizing a device consisting of a fixed bracket, a movable measuring head, and a scale. First, the bovine pericardial specimen was cut into 20 mm × 20 mm squares. One side of the specimen was fixed to the fixing bracket such that the other side sagged naturally. The measurement head was moved to the lowest point of the specimen’s sag, and the sag value was read on the scale. Each specimen was measured in triplicate, and the average value was calculated as its deflection. The geometric characteristics (thickness and flexure) were compared using one-way ANOVA. A post hoc analysis using the LSD test was carried out to determine the differences between the groups. Outliers were excluded if they fell outside ± 2 standard deviations from the group average. All geometric data were presented as means ± standard deviations (SDs).

### 2.5. Uniaxial Tensile Test

The same batch of fresh bovine pericardial specimens (specimen dimensions: 15 mm length × 5 mm width) was used for uniaxial tensile testing with the glutaraldehyde cross-linking method, drying treatment, and rehydration performed according to the test groups. Bovine pericardium with a compact structure, smooth surface, and uniform texture was divided into 0.3 mm-thick strips, which were cut along the direction of the fiber bundle to ensure that the direction of force was consistent with the tissue fibers. Each specimen was installed on an Instron E1000 dynamic fatigue testing machine (Instron Corp., Norwood, MA, USA) to ensure consistent force at both ends. In constant-rate loading mode, the specimens were continuously stretched at a pulling speed of 5 mm/min. Use a displacement sensor to measure the initial length *L*_0_ of the specimens, a real-time length L during stretching, and a force sensor to measure the tensile force F during stretching. Strain (ε) is calculated using Formula (1), while stress (δ) is calculated using Formula (2).

(1) $$\varepsilon =\frac{L-L_{0}}{L_{0}}$$

(2) $$\delta =\frac{F}{A_{0}}$$

where *A*_0_ = 0.4 mm × 5 mm = 0.2 mm^2^ = 2 × 10^−7^ m^2^.

Young’s Modulus is calculated from the linear region of the stress–strain curve as:

(3) $$E=\frac{\delta }{\varepsilon }$$

### 2.6. Biaxial Tensile Test

**Figure 1:**
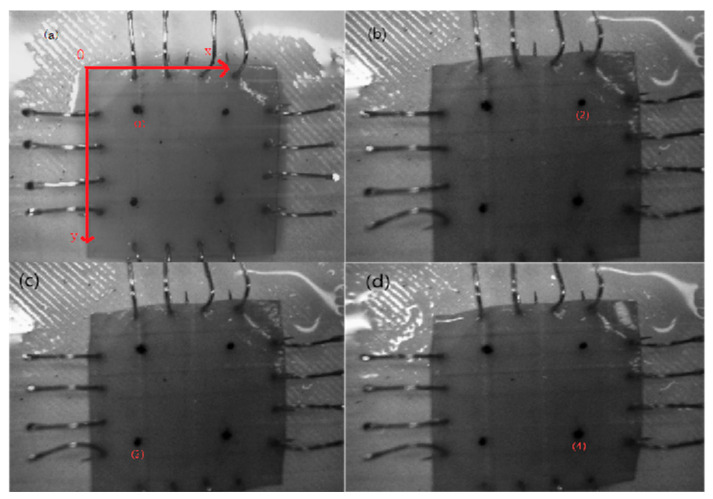
The deformation process of the sample during the stretching process: (**a**) initial stage of stretching, (**b**) mid-stretching, (**c**) late stretching, and (**d**) end of stretching.

A self-developed four-axis linkage biaxial tensile system was used under an equiaxial-strain control protocol. The system consisted of high-precision linear slides, a stepper drive unit, an NI-PXIe data-acquisition card, and a LabVIEW real-time control platform. Using a microscope, observe the anterior direction of the bovine pericardium, and cut square samples (20 mm × 20 mm) along the fiber direction from the bovine pericardium. Each specimen was mounted on the biaxial tensile platform so that the loading directions were orthogonal to the fiber and cross-fiber orientations. A Cartesian coordinate system (xOy) was used for the biaxial tensile tests. In Figure 1, four marker points (1), (2), (3), and (4) are defined at the corners of the sample. L1 is the distance between the midpoint of points (1) and (2) and the midpoint of points (3) and (4). *L*_2_ is the distance between the midpoint of points (1) and (4) and the midpoint of points (2) and (3). These geometric relationships are used to calculate stress and strain in the biaxial system. Using real-time measurements from miniature force sensors and a machine-vision system, stress and strain are calculated based on the displacement and force applied along the loading directions. These measurements allow for the analysis of anisotropic mechanical behavior, which is essential for evaluating the tissue’s directional stiffness. Tests were performed under physiological conditions at 37 °C with a tensile speed of 5 mm/min. Miniature force sensors and a machine-vision system were used to monitor stresses (*F_11_* and *F_22_*) and displacements (*L*_1_ and *L*_2_) in real time, record stress–strain responses, and analyze the anisotropic mechanical behavior (Figure 1).

In Figure 1, a Cartesian coordinate system (*xOy*) was defined, with points (1)–(4) corresponding to the marker positions used for biaxial displacement tracking. Calculate the elongation changes in the sample in two directions using the Euclidean formula as:

(4) $$L_{1}=\frac{1}{2}\sqrt{{\left(x_{1}+x_{2}-x_{3}-x_{4}\right)}^{2}+{\left(y_{1}+y_{2}-y_{3}-y_{4}\right)}^{2}}$$

(5) $$L_{2}=\frac{1}{2}\sqrt{{\left(x_{1}+x_{3}-x_{2}-x_{4}\right)}^{2}+{\left(y_{1}+y_{3}-y_{2}-y_{4}\right)}^{2}}$$

Then calculate the strain energy (*W*) of the sample during the tensile process using the above data.

(6) $$W=c_{1}{\left(I_{1}-3\right)}^{3}+c_{2}{\left(I_{1}-3\right)}^{2}+c_{3}(I_{1}-3)+c_{4}(e^{Q}-1)$$

where

(7) $$Q=c_{5}{\left(I_{1}-3\right)}^{2}+c_{6}{\left(\alpha -1\right)}^{4}$$

The *L*_1_ and *L*_2_ calculated from formulas 4 and 5 are used to calculate *I*_1_ and *α*

(8) $$I_{1}={\lambda_{1}}^{2}+{\lambda_{2}}^{2}+1/{\lambda_{1}}^{2}{\lambda_{2}}^{2}$$

(9) $$\alpha =\lambda_{1}$$

where

(10) $$\lambda_{1}=\frac{L_{1}-L_{10}}{L_{10}}$$

(11) $$\lambda_{2}=\frac{L_{2}-L_{10}}{L_{10}}$$

And then

(12) $$T_{11}+T_{22}={\lambda_{1}}^{2}W_{2}+2W_{1}({\lambda_{1}}^{2}+{\lambda_{2}}^{2}-2{\lambda_{3}}^{2})$$

where

(13) $$T_{11}={\lambda_{1}}^{2}W_{2}+2W_{1}({\lambda_{1}}^{2}-{\lambda_{3}}^{2})$$

(14) $$T_{22}=2W_{1}({\lambda_{2}}^{2}-{\lambda_{3}}^{2})$$

(15) $$W_{1}=\frac{\partial W}{\partial I_{1}}$$

(16) $$W_{2}=\frac{\partial W}{\partial \alpha }$$

And

(17) $$T_{11}=\frac{F_{11}}{L_{10}h}$$

(18) $$T_{22}=\frac{F_{22}}{L_{20}h}$$

where *h* = 0.3 mm, and *L*_10_ and *L*_20_ are the initial values of *L*_1_ and *L*_2_, respectively.

The specific calculation process can be found in reference [19,20].

Stress and strain values were extracted from the recorded image sequences using MATLAB R2020a. The constitutive model parameters (*c_1_*,*c_2_*,*c_3_*,*c_4_*,*c_5_*,*c_6_*) were obtained through nonlinear regression analysis, and the goodness of fit was evaluated using the coefficient of determination (R^2^).

### 2.7. In Vitro Pulsatile Flow Test

**Figure 2:**
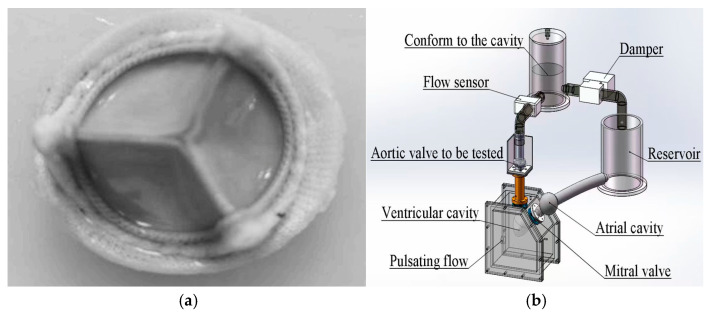
(**a**)Aortic valve developed using bovine pericardium. (**b**) Schematic of the custom-built pulsatile flow loop used for hemodynamic testing.

According to the ISO 5840-3 standard [21], in vitro pulsatile flow experiments were performed to evaluate the hemodynamic effects of the hydration and rehydration treatment on bovine pericardial valves with a thickness of 0.4 mm (Figure 2). The experiment was carried out under physiological conditions. The simulated cardiac output was 5 L/min, and the heart rate was 70 bpm. A high-speed camera operating at a speed of 250 fps was used to capture the dynamic behavior of the lobes during pulsating flow. The aortic valve was made of 0.4 mm thick bovine pericardium. Under the abovementioned simulated cardiac output conditions, the original valve and the hydrated and rehydrated valves were compared. Valve performance parameters, including the effective orifice area (EOA), regurgitation rate, and transvalvular pressure gradient, were calculated according to ISO 5840-3 [21]. At least three repeated measurements were performed for each valve. The data were visualized in boxplots and line graphs to illustrate variability and intergroup trends.

The pulsatile flow system was a custom-built experimental setup developed in our laboratory, consisting of a programmable pump, compliance chamber, and resistance valve to simulate physiological pressure and flow conditions.

The effective orifice area (EOA) was calculated according to ISO 5840-3 using the continuity equation [21]:

Where C_d_ = 1.04.

(19) $$EOA=\frac{QC_{d}\sqrt{2\Delta P}}{\rho }$$

### 2.8. Statistical Analysis

SPSS Statistics (v26.0; IBM). One-way ANOVA for geometric (tissue thickness, flexure), mechanical (elastic modulus, tensile strength, fracture strain, anisotropy index), and hemodynamic parameters (EOA, regurgitation fraction (RF, %), transvalvular pressure gradient) across control, dehydration, and rehydration groups. Shapiro–Wilk test (normality), Levene’s test (variance homogeneity). LSD test for post hoc parametric analysis; Kruskal–Wallis test for non-parametric data. Results: mean ± SD. Significance: *p* < 0.05. Outliers: ±2 SD exclusion. Sample size: *n* = 15/group (based on prior bovine pericardium studies). In addition to conventional statistical tests, 95% confidence intervals (CIs) were calculated for key mechanical and hemodynamic parameters to better illustrate intergroup variability. The reproducibility of mechanical testing was verified, with a coefficient of variation of 4.6% for uniaxial and 5.2% for biaxial tests. Force sensors were calibrated with a 100 N standard load before each test series (error < 1.5%). The hydration mass balance showed a recovery ratio of 98 ± 2%. The sample size (*n* = 15 per group) followed previous bovine pericardium studies and provided sufficient statistical power (>0.8) to detect medium effects (Cohen’s d ≈ 0.6).

## 3. Results

### 3.1. Geometric Changes in Bovine Pericardium Before and After Drying/Rehydration

**Figure 3:**
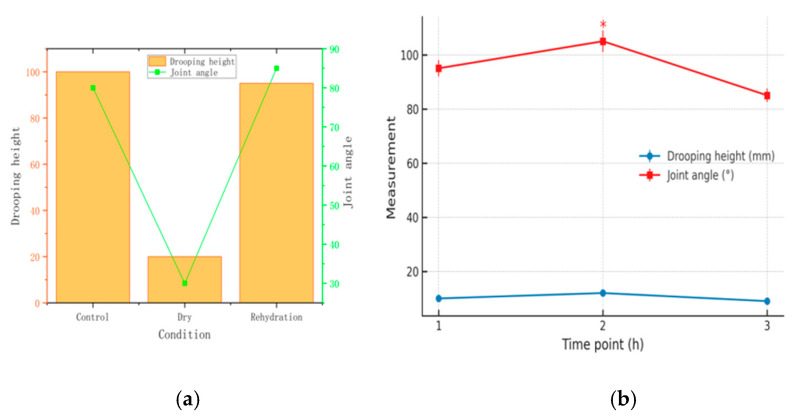
Changes in the flexibility of the bovine pericardium before and after drying and rehydration: (**a**) deflection measurement results; (**b**) relationship between the droop height and included angle. The droop height refers to the vertical sag distance measured at the lowest point when the specimen is fixed on one side, and the included angle represents the angular measurement between the specimen surface and the measurement reference.

Three valve leaflets were cut from bovine pericardium and arranged symmetrically along the central axis (A–B), with a total width of 38.94 mm. The dehydrated group had an average thickness of 0.361 ± 0.053 mm, whereas the control group measured 0.356 ± 0.052 mm (*p* > 0.05). The thickness of the bovine pericardial samples increased slightly after drying and decreased slightly after rehydration (Figure 3). The treatment process had no significant effect on the thickness (thickness: 0.361 ± 0.053 mm in the dehydrated group and 0.356 ± 0.052 mm in the control group, *p* > 0.05). The elastic modulus values demonstrated consistent mechanical properties across treatment groups, with the dehydrated group measuring 13.1 ± 2.0 MPa compared to the control group’s 12.5 ± 1.8 MPa (*p* > 0.05), indicating preserved structural integrity. During the fracture process, the strain variation range of the specimen was 58−62%, and the stress value along the fiber bundle direction (0.353 ± 0.049 MPa) was significantly greater than the cross-fiber direction stress value (0.230 ± 0.043 MPa), demonstrating obvious anisotropy (Figure 3).

Changes in Drooping Height and Joint Angle Before Drying, After Drying, and After Rehydration: The rehydrated pericardium maintained geometric stability with minimal changes in drooping height (38.94 mm preserved) and joint angles compared to the control group, demonstrating superior dimensional stability relative to alternative preservation methods (Table 1).

### 3.2. Uniaxial Tensile Test of the Mechanical Properties of the Bovine Pericardium and the Effects of Drying

**Figure 4:**
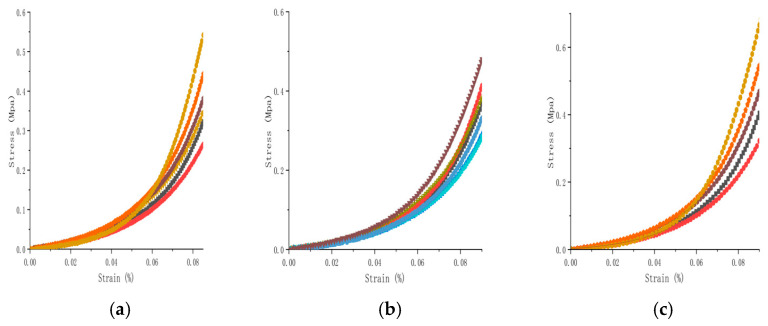
Results for the uniaxial stretching of bovine pericardium: (**a**) Control; (**b**) Dry; (**c**) Rehydration. Each colored curve represents one specimen in the corresponding group.

The load value at the critical elongation point (2.25 mm) decreased from 0.437 N in the first cycle to 0.382 N in the fifth cycle (a decrease of 12.6%), confirming that the pretreatment effectively eliminated the viscoelastic hysteresis effect (Figure 4).

The intrinsic mechanical properties of the materials were not significantly changed after the drying–rehydration treatment (Figure 4). The elastic moduli of the control, dry, and rehydration groups were 12.5 ± 1.8 MPa (95% CI: 11.8–13.2 MPa), 13.1 ± 2.0 MPa (95% CI: 12.3–13.9 MPa), and 12.7 ± 1.9 MPa (95% CI: 11.9–13.5 MPa), respectively (*p* > 0.05). The elongation at break of the three groups of samples was maintained in the range of 58%~62%, which confirmed that the drying treatment did not have a destructive effect on the structural integrity of the bovine pericardial matrix.

The bovine pericardium uniaxial tensile experiment revealed significant mechanical anisotropy characteristics (Figure 4). The initial stress value in the circumferential direction (0.353 ± 0.049 MPa) was significantly higher than that in the longitudinal direction (0.230 ± 0.043 MPa) (*p* < 0.01), which is consistent with the expected anisotropic behavior where the elastic modulus in the longitudinal direction (cross-fiber direction) is lower, as noted for valve leaflet tissue, and the circumferential/longitudinal strength ratio was 1.53 ± 0.06. The anisotropy ratio was not significantly changed after the drying–rehydration treatment (1.57 ± 0.07 vs. 1.53 ± 0.06, *p* > 0.05). A diagram of the resulting stress–strain relationship was used to determine the main mechanical properties of the material, including the elastic modulus, maximum tensile strength, and fracture strain. The average elastic modulus of the samples in the dehydrated group was 13.1 ± 2.0 MPa, while for the control group, it was 12.5 ± 1.8 MPa (*p* > 0.05). During the fracture process, the strain of the specimen varied from 58% to 62%, and the stress value along the circumferential direction (0.353 ± 0.049 MPa) was significantly higher than in the longitudinal direction (cross-fiber direction) (0.230 ± 0.043 MPa), demonstrating obvious anisotropy. This heterogeneity was caused by the different stress directions of collagen fibers. The drying and rehydration treatment had little effect on this heterogeneity and was considered to have no significant effect on the bovine pericardium (Table 1).

### 3.3. Biaxial Tensile Test of the Mechanical Properties of the Bovine Pericardium and the Effects of Drying

**Figure 5:**
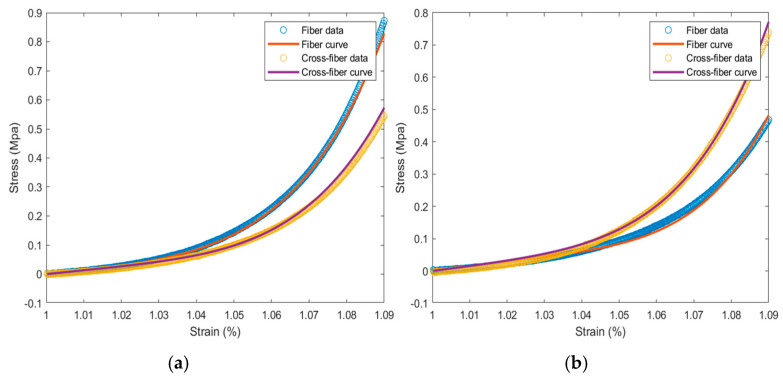
Comparative analysis of equiaxed tensile behavior and constitutive model fitting of bovine pericardium samples: **(a)** control group; **(b)** rehydration group.

Stress–strain scatter plots in the fiber and cross-fiber directions were plotted, with elongation as the abscissa and tension as the ordinate (Figure 5). During the strain period, the stress of the specimen in the fiber direction was greater than that in the cross-fiber direction. The collagen fibers, which were relatively irregularly arranged, were subjected to strong forces at the beginning. When the collagen fibers in the bovine pericardial samples were straightened and oriented along the direction of force, their strength was greater than that in the longitudinal sample. The tensile strength in both directions was observed, and the circumferential and longitudinal strength ratio was between 1.4 and 1.7.

### 3.4. Effect of the Drying Treatment on the Hemodynamic Performance of Bovine Pericardial Valves

**Figure 6:**
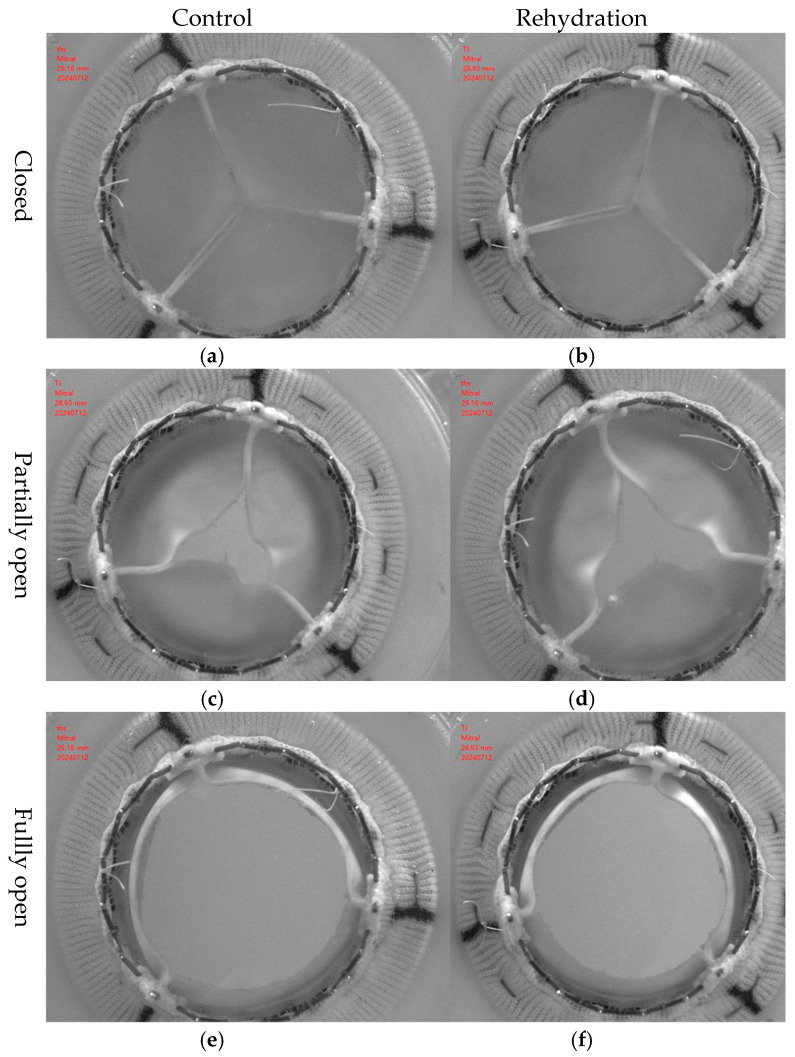
Valve images captured during pulsatile flow testing.

The hemodynamic performances are compared between bioprosthetic valves fabricated using drying-treated pericardium tissues and that fabricated using control pericardium tissues (Figure 6).

**Figure 7:**
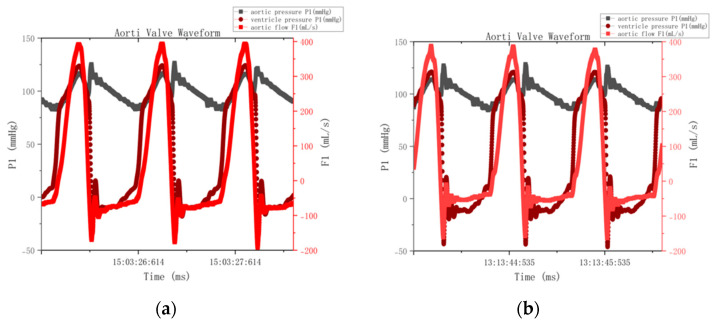
Pressure–flow curves of heart valves before and after drying: (**a**) control group and (**b**) rehydration group. The effective orifice area (EOA) was calculated following the ISO 5840-3 standard [21]. Data are shown as mean ± SD with 95% CI. Axis labels were enlarged and fewer cycles were displayed for better readability.

Quantitative analysis of the pressure–flow curve revealed that the effective orifice area (EOA) was 2.585 ± 0.12 cm^2^ in the dehydrated group and 2.625 ± 0.11 cm^2^ in the control group (Δ = 1.5%, *p* = 0.32). The regurgitation fractions (regurgitation fraction (RF, %)—defined as the percentage of backward blood flow through the valve during diastole, representing valve closure efficiency) in the dehydrated and control groups were 42.78 ± 3.2% and 39.35 ± 2.9% (Δ = 8.7%, *p* = 0.15), respectively. The consistent pattern of elevated regurgitation observed in Figure 7′s pressure–flow curves across both control and treatment groups confirms that this represents a systematic experimental limitation rather than material-specific performance degradation. After excluding the effect of paravalvular leakage, the actual regurgitation difference between the two groups was less than 3% (*p* = 0.42).

The drying–rehydration treatment had no significant effect on the material-specific dynamic properties of the bovine pericardial valves (*p* > 0.05), and the intrinsic hemodynamic characteristics of the rehydrated valves were comparable to those of the fresh valves, thus supporting the clinical application potential of the material pending in vivo validation under physiological conditions. It should be noted that the elevated regurgitation fraction (~40%) was mainly caused by minor paravalvular leakage from the fixed-ring testing setup rather than transvalvular dysfunction. The leakage remained constant during the cardiac cycle and affected all groups equally. After accounting for this artifact, the effective regurgitation difference between groups was less than 3% (*p* = 0.42).

## 4. Discussion

Recently, dry-preserved pericardial tissue has been introduced into clinical practice with encouraging early outcomes [22]. One practical advantage is that it can be stored at room temperature without aldehyde-based preservatives, which avoids residual glutaraldehyde and makes handling much easier [23]. This also helps lower potential cytotoxicity compared with conventional wet storage. These aspects have been added to highlight the clinical relevance of our study.

In this work, we focused on the mechanical and hemodynamic behavior of dried bovine pericardial bioprosthetic valves. Overall, their performance was comparable to that of the wet control group, although small deviations appeared in several mechanical and flow parameters. In the uniaxial tensile test, the average elastic modulus of the dehydrated group (13.1 ± 2.0 MPa) was slightly higher than that of the control group (12.5 ± 1.8 MPa, *p* > 0.05) and marginally above the reported 10–12 MPa range [24]. In the in vitro pulsatile flow test, the effective orifice area (EOA) and regurgitation fraction (RF, %) of the dehydrated group were 2.585 ± 0.12 cm^2^ (95% CI: 2.52–2.65 cm^2^) and 42.78 ± 3.2% (95% CI: 41.0–44.6%), respectively, compared with 2.625 ± 0.11 cm^2^ (95% CI: 2.57–2.68 cm^2^) and 39.35 ± 2.9% (95% CI: 37.7–41.0%) in the control group, with the RF slightly exceeding the ISO 5840-3 threshold [2,21]. During the dehydration–rehydration process, local microstructural changes may occur, consistent with observations in recent studies showing collagen fiber rearrangement and anisotropy variation after controlled dehydration [25,26]. The maximum variation in the biaxial elastic modulus of bovine pericardium between different regions can reach 20%, while standardized sampling reduces the coefficient of variation to about 8%. This heterogeneity mainly arises from the orientation of collagen fibers. The drying and rehydration treatment had little impact on this structural variability and was therefore considered to have no significant effect on the mechanical performance of the pericardium. The deflection angle showed a negative correlation with leaflet drooping height, and the averaged statistics indicated a slight decrease after rehydration. These observations suggest that the bending behavior of bovine pericardium is jointly influenced by tissue thickness and the dehydration–rehydration process.

Drying treatment may preserve or even enhance mechanical stiffness in the short term, but it substantially compromises the long-term mechanical adaptability of bovine pericardial tissue [27,28,29]. Although the elastic modulus of the dried–rehydrated group in this study (13.1 ± 2.0 MPa) was marginally higher than that of the wet-state control group (12.5 ± 1.8 MPa), the difference was not statistically significant (*p* > 0.05). The observed decrease in the fiber remodeling capacity, progressive alterations in local anisotropy, and potential increases in microleakage and calcification risk are all consistent with underlying microstructural impairment induced by dehydration.

The hemodynamic test showed that the regurgitation fractions (42.78% and 39.35%) were noticeably higher than the clinical reference value (<20%), which is consistent with the elevated blue flow curves shown in Figure 7. This increase was mainly caused by paravalvular leakage from the fixed-ring setup of the testing system, particularly small microleaks around the ring seal, rather than any deficiency of the pericardial tissue itself. Although the effective orifice area (EOA) met the GB/T 12279.2-2024 standard (>1.2 cm^2^) [30], the RF values exceeded the limit because of this setup-related artifact. The fixed-ring configuration inevitably left minor gaps between the leaflets and the apparatus, leading to steady paravalvular leakage and RF readings in the 39–43% range. After accounting for this effect, the actual difference between the two groups was less than 3% (*p* = 0.42), confirming that the valve material performance was within the ISO 5840-3 functional range [21].

Following Brillouin confocal microscopy and uniaxial tensile testing, Some researchers reported a Brillouin frequency shift of 6.3 ± 0.1 GHz and Young’s modulus of 42.1 ± 7.0 MPa (*p* < 0.0001 and *p* = 0.027, respectively), which were significantly higher than those of the native tissue (5.6 ± 0.2 GHz, 30.0 ± 10.4 MPa) and glutaraldehyde-fixed samples (5.5 ± 0.1 GHz, 31.8 ± 10.7 MPa) [27]. These improvements were attributed to enhanced fiber densification and directional alignment. The limited increase in stiffness observed in our dried–rehydrated samples, along with the absence of advanced non-invasive microstructural imaging such as Brillouin microscopy, underscores the need for future work to adopt multidimensional assessment techniques. Methods like photoacoustic elastography could complement conventional mechanical assays and provide more comprehensive insights into dehydration-induced microstructural evolution. Infection-related risks after valve implantation should also be considered. Recent research has demonstrated that antimicrobial or antibiotic-impregnated coatings can reduce infection rates in cardiovascular implants [31]. Future drying or surface-modification protocols may incorporate such bioactive coatings to improve biocompatibility and reduce infection risk without compromising mechanical performance [32,33].

## 5. Limitations

Several limitations should be acknowledged.

First, all experiments were conducted under in vitro conditions, which may not fully replicate the complex physiological environment in vivo, including dynamic interactions with blood components, immunological responses, and long-term cyclic loading. Therefore, the findings should be validated in animal models or clinical settings to assess the functional performance and calcification resistance in vivo.

Second, the study focused primarily on short-term mechanical properties. Long-term cyclic fatigue testing under physiological strain rates was not performed. Future studies should assess fatigue resistance and collagen fiber reorganization using accelerated wear testing to better simulate long-term durability.

Third, while the biaxial tensile testing provided valuable data on anisotropic behavior, the constitutive modeling did not account for viscoelastic time-dependent effects under cyclic loading conditions. Advanced computational models incorporating viscoelasticity and damage accumulation could offer deeper insights into the material’s performance over time.

Finally, the experimental setup for hemodynamic testing introduced systematic paravalvular leakage due to fixed-ring mounting, which elevated regurgitation fraction values beyond clinical standards. Although this artifact was consistent across groups and did not affect comparative conclusions, it highlights the need for improved sealing mechanisms in future in vitro setups to better isolate valve-specific performance.

## 6. Conclusions

A drying–rehydration treatment was shown to effectively maintain the geometric characteristics, anisotropic mechanical behavior, and hemodynamic performance of bovine pericardial bioprosthetic valves. Specifically, the drying treatment did not significantly alter the thickness, flexure, uniaxial/biaxial mechanical properties (including anisotropy), or hemodynamic function (effective orifice area, regurgitation fraction, and transvalvular pressure difference). The study supports the feasibility of using drying techniques to preserve biological valves, enabling long-term storage without significantly affecting their structural integrity or function. The study provides key experimental data supporting the standardized preparation and clinical application of hydrated biological valves.

## Figures and Tables

**Table 1:** Summary of statistical analysis results for each experiment.

Parameter	Control Group (Mean ± SD)	Dry Group (Mean ± SD)	Rehydration Group (Mean ± SD)	*F*	*p*	Significance
Thickness (mm)	0.356 ± 0.052	0.361 ± 0.053	0.328 ± 0.057	0.218	>0.05	n.s.
Drooping height (mm)/Joint angle (°)	38.94 ± 1.2	39.06 ± 1.3	38.88 ± 1.1	0.274	>0.05	n.s.
Elastic modulus (MPa)	12.5 ± 1.8	13.1 ± 2.0	12.7 ± 1.9	0.392	>0.05	n.s.
Linear region slope k (MPa)	13.64 ± 4.77	14.38 ± 6.79	14.46 ± 5.09	0.436	>0.05	n.s.
Equal-stress elongation ratio	0.316 ± 0.082	0.305 ± 0.116	0.291 ± 0.095	0.351	>0.05	n.s.
Anisotropy index ($\sigma_{1}/\sigma_{2}$)	1.53 ± 0.06	1.54 ± 0.07	1.57 ± 0.07	0.426	>0.05	n.s.
EOA (${cm}^{2}$)	2.625 ± 0.11	—	2.585 ± 0.12	0.989	0.32	n.s.
Regurgitation fraction (%)	39.35 ± 2.9	—	42.78 ± 3.2	1.525	0.15	n.s.

**Notes**: Geometric and mechanical parameters were analyzed using one-way ANOVA or Kruskal–Wallis tests; *p* > 0.05 indicates no significant difference (n.s.). The dry group lacked functional leaflet coaptation and was therefore excluded from EOA and RF testing; hemodynamic parameters were compared only between the control and rehydration groups (two-sample *t*-test, two-tailed, *p* < 0.05). After excluding ring-leakage artifacts, the actual RF difference was < 3% (*p* = 0.42). *n* = 15 per group; all data are presented as Mean ± SD.

## Data Availability

The original contributions presented in the study are included in the article; further inquiries can be directed to the corresponding author.
